# ASK “to be, or not to be?”

**DOI:** 10.18632/oncotarget.6105

**Published:** 2015-10-13

**Authors:** Tomohiko Okazaki

**Affiliations:** Laboratory of Molecular Biology, Graduate School of Pharmaceutical Sciences, The University of Tokyo, Tokyo, Japan

**Keywords:** innate immunity, viral infection, type I interferon, apoptosis

“To be, or not to be? That is the question.” William Shakespeare, The Tragedy of Hamlet, Prince of Denmark (1599-1602). Viral infection triggers host defense mechanisms that include the production of type I interferon (IFN) and the induction of apoptosis. Type I IFN, such as IFN-α and IFN-β, establishes the antiviral state to inhibits viral replication within infected cells, whereas apoptosis of infected cells altruistically prevents viral propagation to neighboring uninfected cells. Although both of these responses appear to be effective strategies to attenuate viral infection, they can also be detrimental to the host organism in some contexts. It therefore seems likely that host cells would have developed means to differentially regulate type I IFN production and apoptosis in a context-dependent manner so as to maximize the benefits and minimize the costs of these responses to the host organism. Whether or how these two antiviral strategies are indeed regulated differentially has remained unclear, however. In a recent study published in *Science Signaling* [1], we found that the apoptosis signal-regulating kinase (ASK) family of protein kinases determines a cell's decision to produce type I IFN or to undergo apoptosis in response to viral infection.

**Figure 1:**
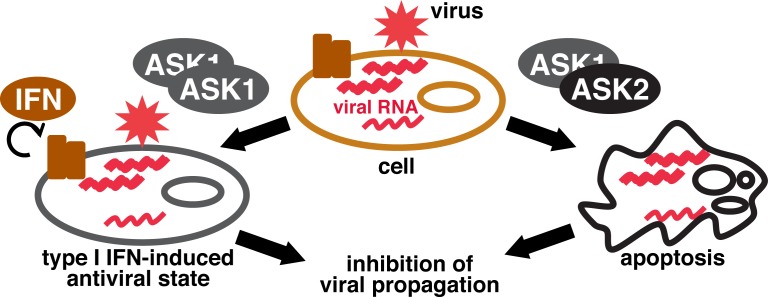
Schematic overview of the ASK family kinases mediated antiviral strategies

We first focused on the mechanism by which cytosolic sensors of viral RNA—the retinoic acid-inducible gene I (RIG-I)-like helicase receptors (RLRs) RIG-I and melanoma differentiation-associated gene 5 (MDA5)—activate the mitogen-activated protein kinases (MAPKs) p38 and c-Jun NH_2_-terminal kinase (JNK) to induce expression of the IFN-β gene. We found that the MAPK kinase kinase (MAPKKK) ASK1 is activated by cytosolic double-stranded RNA and plays an essential role in the induction of both IFN-β production and apoptosis. Infection of ASK1 knockout mice with influenza A virus further revealed that ASK1 is required to suppress viral replication in the lung, suggesting that ASK1 is a novel component of the RLR signaling pathway. We next examined how cells differentially trigger these two ASK1-mediated responses, focusing on the MAPKKK ASK2, which forms hetero-oligomers with ASK1 and modulates ASK1-mediated signaling [2]. By infecting ASK2 knockout mice with influenza A virus, we found that ASK2 is essential for the ASK1-dependent induction of apoptosis but not for type I IFN production. ASK2 was also shown to be required for suppression of viral propagation in the lung. These findings thus suggested that ASK2-dependent apoptosis is a key antiviral strategy in this system. Given that ASK2 forms hetero-oligomers with ASK1 but does not form homo-oligomers, ASK1-ASK2 hetero-oligomers may mediate apoptosis, whereas ASK1 homo-oligomers mediate the production of type I IFN (Figure 1). How might ASK1 homo-oligomers and ASK1-ASK2 hetero-oligomers trigger such different outputs given that these two proteins belong to the same family and share many structural features [3]? One possible explanation is that ASK2 preferentially activates JNK, the sustained activation of which leads to apoptosis, rather than p38 [2, 4]. It is also possible that ASK1 and ASK2 each have specific downstream targets that are regulated independently of MAPK activation. Further studies are needed to investigate these possibilities.

Apoptosis is a two-edged sword in that it removes cells that are infected but which may also be needed by the host, especially if they are in short supply. The benefits of apoptosis outweigh the risks, however, if the particular cell type targeted by the virus is plentiful, such as epithelial cells in epithelium-rich tissues. Intriguingly, whereas ASK1 appears to be ubiquitously expressed, ASK2 is highly abundant in epithelium-rich tissues with a rapid repair rate such as lung and skin, but not in non-epithelium-rich tissues such as brain and heart [5]. We thus propose that epithelial cells with a rapid repair rate efficiently eliminate viruses through ASK2-dependent apoptosis, whereas other cell types with a slow repair rate maintain tissue homeostasis by eliminating viruses through ASK1-dependent production of type I IFN. In other words, the abundance of ASK2 may be a key determinant of whether virus-infected cells decide to commit suicide or not-reminiscent of the traveling troupe that made Hamlet decide to exact revenge at the risk of losing his own life (to be, or not to be). Type I IFN is also not always beneficial to the host organism, and indeed can be harmful under some circumstances. It has thus been found to have deleterious effects in certain bacterial infections [6] and to reduce the number of hematopoietic stem cells [7]. Whether ASK2-dependent apoptosis is beneficial in these contexts is therefore worthy of future study. In summary, our findings reveal a new framework of cellular decision-making, addressing how host cells discriminate between different strategies in their response to environmental stimuli as well as the consequences of blockade of such discrimination.
